# Outdoor biting by *Anopheles* mosquitoes on Bioko Island does not currently impact on malaria control

**DOI:** 10.1186/s12936-015-0679-2

**Published:** 2015-04-21

**Authors:** John Bradley, Jo Lines, Godwin Fuseini, Christopher Schwabe, Feliciano Monti, Michel Slotman, Daniel Vargas, Guillermo Garcia, Dianna Hergott, Immo Kleinschmidt

**Affiliations:** MRC Tropical Epidemiology Group, London School of Hygiene and Tropical Medicine, London, UK; Department of Disease Control, London School of Hygiene and Tropical Medicine, London, UK; Medical Care Development International, Malabo, Equatorial Guinea; Medical Care Development International, Silver Spring, MD USA; Texas A & M University, College Station, TX USA; Faculty of Health Sciences, University of the Witwatersrand, Johannesburg, South Africa

## Abstract

**Background:**

There have been many recent reports that the rate of outdoor biting by malaria vectors has increased. This study examined the impact this might have on malaria transmission by assessing the association between exposure to outdoor bites and malaria infection on Bioko Island, Equatorial Guinea.

**Methods:**

Responses to questions about time spent outside the previous night from a malaria indicator survey were combined with human landing catch measurements of hourly rates of outdoor and indoor biting for the whole island to estimate the number of outdoor and indoor bites received by each survey respondent. The association between RDT measured malaria infection status of individuals and outdoor bites received was investigated.

**Results:**

The average number of bites received per person per night was estimated as 3.51 in total, of which 0.69 (19.7%) would occur outdoors. Malaria infection was not significantly higher in individuals who reported spending time outside between 7 pm and 6 am the previous night compared to those not spending time outside in both adults (18.9% *vs* 17.4%, p = 0.20) and children (29.2% *vs* 27.1%, p = 0.20). Malaria infection in neither adults (p = 0.56) nor in children (p = 0.12) was associated with exposure to outdoor bites, even after adjusting for confounders.

**Conclusions:**

Malaria vector mosquitoes in Bioko do bite humans outdoors, and this has the potential to reduce the effectiveness of vector control. However, outdoor biting is currently not a major factor influencing the malaria burden, mainly because more than 95% of the population are indoors during the middle of the night, which is the peak biting period for malaria vector mosquitoes. The majority of resources should remain with control measures that target indoor biting and resting such as LLINs and IRS.

## Background

African malaria vectors have a strong tendency to bite humans sleeping indoors [1-4]. However, in recent years there have been reports of these mosquitoes biting humans outdoors more frequently, on Bioko [5] and elsewhere in Africa [6]. In this study the effect this behaviour might have on the risk of malaria infection was investigated.

If malaria vectors are biting more outdoors this could be as a result of intensive efforts to control them over the last decade [7]. The majority of this scale-up in vector control comes in the form of interventions which target mosquitoes indoors: long-lasting insecticidal nets (LLINs) and indoor residual spraying (IRS). The effectiveness of LLINs depends on vectors biting humans while they sleep (i.e. mostly indoors), while the effectiveness of IRS depends on vectors resting indoors. Vector control interventions that target indoor biting vectors can cause such apparent shifts in behaviour in at least three ways. The first is excito-repellency: this is a short-lived phenotypic effect of exposure to sub-lethal doses of insecticide. The second is a shift in sibling species ratio, as for example when two sibling species are both reduced in abundance by an intervention, but one is reduced more than the other because it is more likely to bite indoors [8]. The third is genuine behavioural resistance: this happens when there are genetically-determined behavioural variants within a species, and selection pressure by the intervention causes an evolutionary shift in the frequency of these variants, so that those less affected by the intervention become more common [3]. Behavioural changes due to excito-repellency are less of a long-term threat to control because they are phenotypic and temporary and not progressive, whereas those that are genetically-controlled and evolved are much more dangerous, because they are long-term, hard to reverse, and potentially progressive.

Since the 1950s, outdoor biting (exophagy) and outdoor resting (exophily) have been recognized as important factors reducing the effectiveness of IRS and its capacity to interrupt transmission [4]. It has been argued that because of exophagy and exophily additional interventions may be necessary to maintain reductions in malaria transmission and to achieve malaria elimination [2,4,9]. While numerous entomological studies [6,10-13] and mathematical models [3,10] have investigated the impact of exophagy, little evidence has been published linking outdoor biting to epidemiological malaria outcomes.

If outdoor biting is a major factor in malaria transmission on Bioko one would expect that people who spend time outdoors at times when *Anopheles* mosquitoes bite humans would be at greater risk of malaria than those who do not. In this study, entomological data were combined with parasitological data from a 2013 malaria indicator survey, to estimate respondents’ exposure to outdoor biting in relation to their risk of malaria infection.

## Methods

### Study area

Bioko, the main island of Equatorial Guinea, has a population of approximately 250,000 and is situated 32 kilometres off the coast of Cameroon. Malaria transmission occurs throughout the year. The Bioko Island Malaria Control Project (BIMCP), funded by the Government of Equatorial Guinea and a consortium of private donors led by Marathon Oil Corporation, was launched in 2004. The project carried out island-wide IRS, the first round taking place in 2004 with deltamethrin, (K Orthrine WG 250, Bayer). This was followed by biannual rounds of the carbamate insecticide bendiocarb (FicamTM, Bayer) from 2005 to 2012. In 2013 and 2014 a single round of a long-lasting micro-encapsulated formulation of deltamethrin (K Orthrine SC 65, Bayer) was carried out each year. IRS coverage was frequently inadequate; for example the most recent round of IRS in 2014 sprayed 55% of houses with insecticide. In 2005 intermittent preventative treatment for pregnant women (IPTp), case management using artemisinin-based combination therapy (ACT), and rapid diagnostic tests (RDT) together with the training of health facility staff were introduced as additional measures. A mass LLIN distribution took place in 2007 with 110,000 PermaNet 2.0 nets (Vestergaard Frandsen, Lausanne, Switzerland) distributed to over 38,000 households. Initially high levels of LLIN ownership and usage were achieved with 76% of two to 14 year olds reported to be sleeping under an LLIN, but coverage declined rapidly thereafter; in 2013 only 13% of two to 14 year old children were reported to be sleeping under a LLIN [14,15]. Malaria prevalence in two to 14 year old children was 45% before the start of interventions but dropped to 32% in 2005 and 26% in 2006 [16]. After several years of little further change, prevalence declined to 14% in 2012 before rebounding to 28% in 2013. Moderate to severe anaemia (Hg < 8 g/dL) fell from 15% to 2%, and all cause under-five mortality declined from 152 per 1,000 births to 55 per 1,000 in the first four years post intervention [15].

Before the launch of the BIMCP, malaria was transmitted by *Anopheles funestus* and both the S and M molecular forms of *Anopheles gambiae s.s.* and in coastal areas by *Anopheles melas*. High entomological inoculation rates (EIR) of over 750 and 250 infectious bites per person per year by *An. funestus* and *An. gambiae s.s.,* respectively, were recorded [17,18]. After the first three spray rounds no specimens of *An. funestus* were observed and the proportion of S-form *An. gambiae s.s.* was reduced [19]*.* More recent studies found no specimens of either S-form *An. gambiae s.s.* or *An. funestus* [5,11,20]. The relative exophagy of malaria vectors in Bioko, as measured by simultaneous human-landing catches indoors and outdoors, has increased substantially over the years since the start of the BIMCP. Studies conducted before the start of the Project came to the conclusion that outdoor biting, whilst clearly occurring, was unimportant relative to indoor biting, which occurred at rates of up to 80 bites per hour at peak times [17,18]. By 2009 however, the outdoor biting rate by *An. gambiae s.s* was 8.4 bites per person per hour, compared to an indoor biting rate of 5.9 [5,11].

### Epidemiological monitoring

Since the start of the BIMCP in 2004 annual household malaria indicator surveys have been conducted on Bioko [14-16,21,22]. Monitoring the impact of the BIMCP is based on a system of eighteen sentinel sites, of which five are in Malabo (urban), five are peri-urban and eight are rural. A random sample of houses was taken at each site using household lists compiled by the Project. The survey instrument was adapted from the standard malaria indicator survey developed by the Roll Back Malaria Monitoring and Evaluation Reference Group [23]. Sample size was determined to show a change in prevalence of infection from 20% to 17% between years, assuming a design effect of 2.5. This study is based on the 2013 survey in which all members of a sampled household who were present had their haemoglobin measured (HemoCue, Ängelholm, Sweden) and were tested for *Plasmodium falciparum* using RDT (Carestart, AccessBio Inc., Monmouth, USA), subject to informed written consent (from a caregiver in the case of children). Those testing positive for parasitaemia were provided with ACT (artesunate + amodiaquine) by a Project nurse. Those with haemoglobin < 8 g/dL or who were febrile were referred to a local clinic for appropriate treatment (anti-malarial, anti-pyretic, or iron supplementation). Participants were asked at what time they entered the house the night before the survey, about any other time spent outside the house between 7 pm and 6 am and what time they went to bed. Behaviour during the previous night was taken as a proxy for usual behaviour, in the same way that bed net use the previous night is asked as a proxy for usual behaviour.

### Entomological monitoring

Human Landing Catches (HLCs) were carried out monthly from January to October at six sites. These sites were all located within the system of sentinel sites used for the MIS surveys. A team of eight catchers worked through the night from 7 pm to 6 am. Four houses approximately 100 metres apart were selected in each site for conducting HLC throughout the study period. Each house had one catcher inside and another immediately outside, under the veranda if there was one. At midnight the indoor and outdoor catchers exchanged positions. Mosquitoes were stored in collection tubes labeled according to the hour and the location of capture. Two entomology field supervisors were responsible for ensuring that the volunteers stayed awake during the night. Catchers were informed of the risks involved and were offered treatment if they showed symptoms of malaria.

### Statistical analysis

From survey responses, the proportion of participants indoors and outdoors at each hour of the night was calculated for each site and for the island as a whole. These proportions were multiplied by the indoor and outdoor biting rates from HLCs to estimate biting exposure in terms of the indoor and outdoor bites received at each hour of the night between 7 pm and 6 am per Bioko resident. This enabled the estimation of the proportion of *Anopheles* bites received outdoors, and π_i_ which Killeen *et al*. define to be the proportion of bites occurring indoors for an unprotected individual [24]. This proportion was estimated for the Island as a whole and separately for each sentinel site. For each survey respondent, the times at which they were outdoors at night and the biting rate at those times were used to estimate the number of outdoor bites they could have had received the night before the survey.

The association between RDT confirmed malaria infection and the number of outdoor bites received the night before the survey was assessed using logistic regression. Similarly, the effect of site level proportion of outdoor biting on individual malaria infection was investigated. Analyses were done separately for children aged two to14 and for individuals aged 15 or over. Odds ratios (ORs) were adjusted for the following site level confounders: Sero-Conversion Rate (SCR) measured in 2008 as a measure of underlying transmission intensity [25]; and spray coverage. And for the following individual level confounders: age; sex; net use; asset based household Socio-Economic Status (SES) quintile calculated by Principal Component Analysis (PCA); living in a house with closed eaves; and travel to mainland Equatorial Guinea in the eight weeks before the survey [26].

The association between site level proportion of outdoor biting and the site level prevalence of malaria infection in children aged two to14, was examined using linear regression, adjusting for the site level SCR, the proportion of children at the site who had slept under a net the night before the MIS and the proportion of houses at the site which had received IRS in the previous six months. In all analyses robust standard errors were used to account for the survey design [27,28]. All analyses were performed using Stata version 13 [29].

### Ethics and informed consent

Ethics approval for the study was granted by the Equatorial Guinea Ministry of Health and Social Welfare and the ethics committee of the London School of Hygiene and Tropical Medicine (approval number 5556). Informed written consent was given by each survey participant or, in the case of children, a responsible adult. In the case of participants being unable to read, the text was read and explained to them, and consent was confirmed by an independent witness identified on the consent form.

## Results

The rate of indoor biting, estimated by HLCs, decreased from 39.6 bites per person per night to 3.1 over the five years from 2009 to 2013 (Table 1). The rate of outdoor biting also decreased over the same period, from 53.8 bites per person per night to 10.3. Since indoor biting rates fell more dramatically than outdoor, the proportion - but not the absolute number - of mosquitoes caught outside increased over this five-year period.

**Table 1:** **Indoor and outdoor biting rate of** ***Anopheles*** **mosquitoes**

	**2009**	**2010**	**2011**	**2012**	**2013**
**Indoors**	39.6	15.9	3.8	4.4	3.1
**Outdoors**	53.8	19.6	10.7	10.7	10.3
**Percentage outdoors**	58%	55%	74%	70%	77%

Number of *Anopheles* mosquitoes caught per person per night (7 pm – 6 am) indoors and outdoors by human landing catches, and percentage of mosquitoes caught outdoors on Bioko from 2009 to 2013.

A total of 4,721 households and 21,743 individuals were sampled in the 2013 malaria indicator survey. Of these, 14,792 persons were tested for malaria infection using an RDT. Overall 21.6% tested positive for *P. falciparum* infection (n = 3,187). Infection prevalence was 27.8% (1,709/6,159) among children aged two to 14 years and 18.2% (1,330/7,305) among those aged 15 or over. Site-specific prevalence of malaria infection in children ranged from 2.4% to 55.6%.

Sixty three percent of respondents were reported to be indoors between 7 pm and 8 pm the night before the survey took place. This rose monotonically over the course of the night, reaching 97% between the hours of midnight and 1 am and staying at or above this level throughout the night (Table 2). Children aged 2–14 years spent more time indoors than adults; males spent more time outside than females; and people living in urban areas spent more time outdoors at night than those in peri-urban and rural areas.

**Table 2:** **Percentages of survey respondents reporting being at different hours of the night on Bioko in 2013**

	**Proportion indoors, %**							
**Time of night**	**All ages, all sites**	**Children 2 to 14 years**	**Aged 15 years or over**	**Males, all ages**	**Females, all ages**	**Urban sites**	**Peri-urban sites**	**Rural sites**
7-8	63	74	49	57	67	62	64	63
8-9	73	85	60	68	77	71	76	75
9-10	83	93	73	80	86	81	87	85
10-11	90	97	83	88	92	89	92	92
11-12	95	99	91	93	96	94	96	96
12-1	97	100	95	96	98	97	98	98
1-2	99	100	97	98	99	98	99	99
2-3	99	100	98	98	100	99	99	99
3-4	99	100	99	99	100	99	100	100
4-5	99	100	98	98	99	99	99	99
5-6	98	100	96	97	99	98	98	98

**Figure 1:**
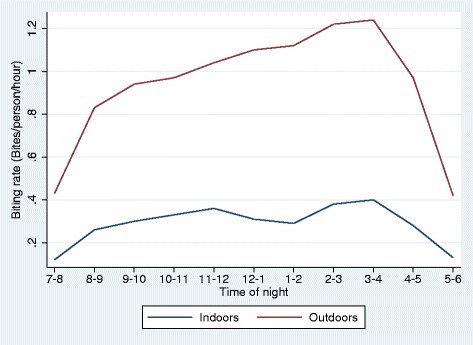
The indoor and outdoor biting rates averaged across Bioko Island in 2013. Biting rates recorded by indoor and outdoor human landing catches in 2013. The y-axis shows the mean number of times per hour a catcher was bitten by an anopheles mosquito.

**Figure 2:**
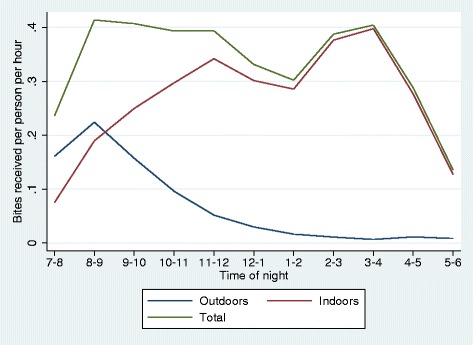
Rate at which the population was bitten indoors, outdoors, and overall on Bioko in 2013. The hourly number of indoor and outdoor bites received per person during each hour of the night, Bioko 2013. The estimated total number of bites received by each Bioko residents in one night is 3.51 (area under green line). Of these, 0.69 (20%) were received outdoors (area under blue line), and 2.82 were received indoors (area under red line).

The outdoor biting rate obtained from HLCs was much higher than the indoor biting rate at all times of the night (Figure 1). However, the time with the highest proportion of people outdoors, 7 pm to 8 pm, was the time with the lowest biting rates. Although rates of outdoor biting can be more than three times those of indoor biting, the location of the population at those times results in more biting occurring indoors than outdoors after about 8 pm (Figure 2). The areas under the curves in Figure 2 represent the average number of bites received per person per night, estimated as 3.51 bites per night in total, of which 0.690 (19.7%) would occur outdoors; therefore π_i_ = 0.803. This assumes that no nets are used. Data from the 2013 MIS indicate that 15.2% of respondents slept under an LLIN the night before the survey and a further 16.2% sleep under an untreated net. Assuming that no bites were received by those sleeping under a net after the reported time of going to bed, then 25.1% of all bites would occur outdoors.

The community percentage of biting taking place outdoors ranged from 13% to 24% between sites, with mean 18.5%. Individuals on average received 0.7 bites per night outdoors (range = 0 to 10.3; median = 0). On average children aged 2–14 years received 0.4 bites per night outdoors whereas persons 15 or over were exposed to 1.3 bites per night outdoors due to their greater outdoor exposure.

Malaria infection was not significantly higher in individuals who reported spending time outside between 7 pm and 6 am the previous night compared to those who did not, in both adults (18.9% *vs* 17.4%, *p* = 0.20) and children (29.2% *vs* 27.1%, *p* = 0.20) (Table 3 and Table 4). Malaria infection in neither adults (*p* = 0.56) nor in children (*p* = 0.12) was associated with exposure to outdoor bites, even after adjusting for confounders. There was no evidence of an association between the proportion of biting occurring outdoors at a site level and individual infection status.

**Table 3:** **Associations between malaria infection and outdoor exposure in children aged 2 – 14 on Bioko in 2013**

		**Infection prevalence %, (N)**	**Odds ratio (95% CI)**	***p*** **-value**	Adjusted odds ratio* (95% CI)	***p*** **-value**
**Child spent any time outside between the hours of 7 pm and 6 am**	No	27.1 (4920)	1	0.20	1	0.87
	Yes	29.2 (1357)	1.11 (0.94 - 1.31)		0.98 (0.82 - 1.17)	
**Estimated number of outdoor bites received by child**	0	27.7 (4122)	1	0.12	1	0.17
	> 0 to <1	30.25 (628)	1.13 (0.90 – 1.44)		1.11 (0.83 – 1.50)	
	≥ 1 to < 4	23.6 (670)	0.81 (0.63 – 1.04)		0.74 (0.55 – 1.01)	
	≥ 4	31.8 (189)	1.22 (0.83 – 1.78)		1.09 (0.61 – 1.94)	
**Site level percentage of bites which occur outdoors**	< 16%	27.7 (775)	1	0.31	1	0.70
	16% to 18%	16.1 (1111)	0.50 (0.14 – 1.73)		0.44 (0.10 – 2.00)	
	18% to 21%	28.6 (2703)	1.04 (0.42 – 2.60)		0.87 (0.19 – 4.12)	
	> 21%	34.5 (1570)	1.37 (0.60 – 3.15)		0.98 (0.20 – 4.93)	

*Adjusted for spray coverage, net use, age, site level SCR, SES, age, sex, sleeping in a house with closed eaves and travelling off the island.

**Table 4:** **Associations between prevalence and outdoor exposure in those aged 15 or over on Bioko in 2013**

		**Infection prevalence %, (N)**	**Odds ratio (95% CI)**	***p*** **-value**	Adjusted odds ratio* (95% CI)	***p*** **-value**
**Individual spent any time outside between the hours of 7 pm and 6 am**	No	17.4 (3100)	1	0.20	1	0.88
	Yes	18.9 (3645)	1.11 (0.94 – 1.31)		1.00 (0.84 – 1.18)	
**Estimated number of outdoor bites received by individual**	0	17.2 (3131)	1	0.56	1	0.41
	> 0 to < 1	17.9 (709)	1.04 (0.77 – 1.44)		0.80 (0.60 – 1.08)	
	≥ 1 to <4	18.5 (1519)	1.09 (0.89 – 1.33)		0.90 (0.70 – 1.16)	
	≥ 4	19.2 (1107)	1.15 (0.94 – 1.40)		0.88 (0.72 – 1.08)	
**Site level percentage of bites which occur outdoors**	< 16%	17.6 (839)	1	0.54	1	0.84
	16% to 18%	14.8 (974)	0.80 (0.27 – 2.39)		0.73 (0.21 – 2.60)	
	18% to 21%	17.5 (3521)	0.98 (0.47 – 2.06)		0.98 (0.30 – 3.20)	
	> 21%	21.5 (1971)	1.22 (0.62 – 2.40)		1.07 (0.33 – 3.51)	

*Adjusted for spray coverage, net use, age, site level SCR, SES, age, sex, sleeping in a house with closed eaves and travelling off the island.

**Figure 3:**
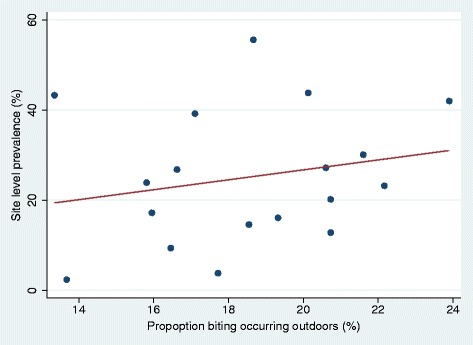
Association between outdoor biting and malaria prevalence by locality. Scatter graph and regression line showing the relation of prevalence of malaria infection in 2 – 14 year-olds and proportion of bites received outdoors at each of the 18 sentinel sites on Bioko in 2013. The slope of the line is 1.10 (95% CI: −1.55 to 3.76, *p* = 0.39), i.e. a 1% increase in the percentage of bites occurring outdoors corresponds to a 1.10% increase in malaria prevalence in children.

There was an estimated trend of a 1.1% increase in site level infection prevalence in children per 1% increase in proportion of bites received outdoors but this was not statistically significant even after adjusting for confounders (Figure 3; *p* = 0.32).

## Discussion

In this study the hypothesis that the risk of malaria in Bioko is associated with human-vector contact outdoors, such that people who spend more time outdoors during peak biting times are at greater risk was examined. No evidence was found that spending time outdoors at night is a risk factor for malaria.

Outdoor biting certainly does take place: human catchers outdoors caught more mosquitoes than those stationed inside (Figure 1). This is in contrast to pre-intervention entomological studies where outdoor biting rates were considered negligible compared to indoor biting rates by investigators [17,18]. It is important to note, though, that this apparent shift is relative: there was a substantial decline in observed biting rates both indoors and outdoors, but the reduction indoors was greater than that outdoors (Table 1).

The reason that outdoor biting is apparently not having a substantial impact on malaria transmission could be that most of the human population is indoors for most of the night. Since most bites from *Anopheles* mosquitoes occur at night they must occur predominantly indoors (Figure 2 and Table 2). In fact, this study may well have underestimated the proportion of bites which occur indoors. This is because many of the mosquitoes which were caught by a catcher outdoors would presumably have come indoors to feed if that catcher had been sleeping indoors along with 97% of the rest of the population. Other studies reporting an apparent shift in vector biting behaviour in response to vector control have also concluded that most transmission still takes place indoors, because that is where most people are at night [1,12].

A previous study on Bioko found no evidence that children who spent time outdoors the night before the survey had higher risks of malaria infection [21]. This study used detailed information about the amount of time spent outside, weighted according to the observed patterns of vector biting rates, to construct a quantitative estimate of outdoor exposure to malaria vectors for each individual. There was no evidence of an association between this measure of outdoor exposure and malaria infection in either children or adults (Tables 3 and 4).

The effect of living in a site with high levels of outdoor exposure was also examined. There was no evidence of an association between the site-specific proportion of the population with outdoor exposure and individual infection status in either adults or children (Tables 3 and 4). Site level infection prevalence and site level outdoor biting exposure showed a very weak and non-significant positive association, after adjusting for potential confounders. The prevalence of malaria infection in children aged two to 14 declined from 45% in 2004 to 23% in 2008 coinciding with a dramatic reduction in indoor biting. Subsequently the prevalence of malaria infection in children aged two to 14 fell to 14% in 2012 while the proportion of mosquitoes caught outside in HLCs had risen to 70% (Table 1). These data suggest that in a high transmission setting, effective malaria control by LLINs and IRS is feasible despite the presence of substantial numbers of blood-seeking mosquitoes outdoors. Similar conclusions have been reached through mathematical modelling [30].

It has been shown before that housing characteristics can affect malaria risk, and future developments in housing quality could have a large impact on malaria transmission on Bioko [14]. House design features that impede mosquito entry are expected to add to the protection of people indoors, but it might also increase the relative importance of mosquitoes biting outdoors.

The data in Table 1 suggest that between 2009 and 2013, there was a substantial decline (about ten-fold) in the density of *Anopheles* mosquitoes biting indoors, and a slightly smaller decline (about five-fold) in the density biting outdoors. The indoor:outdoor biting ratio is presumably influenced not only by the mosquitoes’ intrinsic behavioural characteristics, but also by some environmental factors, especially the room used for indoor biting catches; for example ease of access via the eaves and windows, the presence of an electric light, and the possibility of some unrecorded shift over time in such environmental factors cannot be ruled out. Assuming there was a shift in behaviour, the question would then arise whether this is an evolved heritable change in behaviour and whether there is a genetically-determined subset of mosquitoes that avoid IRS by resting entirely (or almost entirely) outdoors. This would be important because the existence of such mosquitoes has been postulated to explain the limited effectiveness of other malaria vector control programmes [31]. The behaviour of mosquitoes with respect to endophagy and exophagy will form part of the future research agenda of the BIMCP.

A limitation of this study is that the epidemiological data are observational, and although the analysis was adjusted for many observed confounding variables, there could still be residual confounding. This would be a limitation of any such study since it is not possible to randomly allocate communities to different levels of outdoor behaviour. It is standard practice to measure net use by asking whether interviewees used a net the night before the survey, as a proxy for their habitual use of nets [23]. Here the same methodology was used to assess the amount of time individuals spent outside at night, but there are no data on whether activity the previous night is a good proxy for habitual behaviour in this case. In particular, there is anecdotal evidence that people stay out later on Saturday nights in urban areas. Since survey interviews did not take place on Sundays, no data were available for Saturday nights. Adult men are the people most likely to be outside at night (Table 2). They are also the least likely to be at home when the survey is conducted. This potential bias may affect estimates of the proportion of bites which take place outdoors, but not the association between individual exposure to outdoor biting and malaria risk. Another limitation is the assumption of a constant biting rate across the Island. Biting rates, and in particular the relative rates of indoor and outdoor biting, probably vary by locality, but HLCs are a labour-intensive way of collecting entomological data and it was not feasible to perform them at each of the 18 sentinel sites.

## Conclusions

Malaria vector mosquitoes in Bioko do bite humans outdoors, and this has the potential to reduce the effectiveness of indoor vector control. However the results presented here show that it is currently not a major factor influencing the malaria burden, probably because more than 95% of people are indoors during the middle of the night, which is the peak biting period for malaria vector mosquitoes. The results of this study suggest that for the present the majority of resources should remain with control measures that target indoor biting exposure such as IRS and LLINs. To prepare for elimination, however, further research is warranted on potential shifts in vector behaviour, including outdoor biting (e.g. to identify whether an exclusively outdoor biting fraction of *An. gambiae* exists), and on the potential impact of larval source management or personal protective measures during peak periods when the population is outdoors at night.

## References

[1] Huho B, Briet O, Seyoum A, Sikaala C, Bayoh N, Gimnig J (2013). Consistently high estimates for the proportion of human exposure to malaria vector populations occurring indoors in rural Africa. Int J Epidemiol.

[2] Govella NJ, Ferguson H (2012). Why use of interventions targeting outdoor biting mosquitoes will be necessary to achieve malaria elimination. Front Physiol.

[3] Killeen GF, Chitnis N (2014). Potential causes and consequences of behavioural resilience and resistance in malaria vector populations: a mathematical modelling analysis. Malar J.

[4] Pates H, Curtis C (2005). Mosquito behavior and vector control. Annu Rev Entomol.

[5] Reddy MR, Overgaard HJ, Abaga S, Reddy VP, Caccone A, Kiszewski AE (2011). Outdoor host seeking behaviour of *Anopheles gambiae* mosquitoes following initiation of malaria vector control on Bioko Island, Equatorial Guinea. Malar J.

[6] Russell TL, Govella NJ, Azizi S, Drakeley CJ, Kachur SP, Killeen GF (2011). Increased proportions of outdoor feeding among residual malaria vector populations following increased use of insecticide-treated nets in rural Tanzania. Malar J.

[7] WHO. World Malaria Report 2013. World Health Organization, Geneva. http://www.who.int/malaria/publications/world_malaria_report_2013/en/

[8] Bayoh MN, Mathias DK, Odiere MR, Mutuku FM, Kamau L, Gimnig JE (2010). *Anopheles gambiae*: historical population decline associated with regional distribution of insecticide-treated bed nets in western Nyanza Province. Kenya. Malar J.

[9] Russell TL, Beebe NW, Cooper RD, Lobo NF, Burkot TR (2013). Successful malaria elimination strategies require interventions that target changing vector behaviours. Malar J.

[10] Govella NJ, Okumu FO, Killeen GF (2010). Insecticide-treated nets can reduce malaria transmission by mosquitoes which feed outdoors. Am J Trop Med Hyg.

[11] Overgaard HJ, Reddy VP, Abaga S, Matias A, Reddy MR, Kulkarni V (2012). Malaria transmission after five years of vector control on Bioko Island, Equatorial Guinea. Parasit Vectors.

[12] Seyoum A, Sikaala CH, Chanda J, Chinula D, Ntamatungiro AJ, Hawela M (2012). Human exposure to anopheline mosquitoes occurs primarily indoors, even for users of insecticide-treated nets in Luangwa Valley, South-east Zambia. Parasit Vectors.

[13] Stevenson J, St Laurent B, Lobo NF, Cooke MK, Kahindi SC, Oriango RM (2012). Novel vectors of malaria parasites in the western highlands of Kenya. Emerg Infect Dis.

[14] Bradley J, Rehman AM, Schwabe C, Vargas D, Monti F, Ela C (2013). Reduced prevalence of malaria infection in children living in houses with window screening or closed eaves on Bioko Island, equatorial Guinea. PLoS One.

[15] Kleinschmidt I, Schwabe C, Benavente L, Torrez M, Ridl FC, Segura JL (2009). Marked increase in child survival after four years of intensive malaria control. Am J Trop Med Hyg.

[16] Kleinschmidt I, Sharp B, Benavente LE, Schwabe C, Torrez M, Kuklinski J (2006). Reduction in infection with *Plasmodium falciparum* one year after the introduction of malaria control interventions on Bioko Island, Equatorial Guinea. Am J Trop Med Hyg.

[17] Cano J, Berzosa PJ, Roche J, Rubio JM, Moyano E, Guerra-Neira A (2004). Malaria vectors in the Bioko Island (Equatorial Guinea): estimation of vector dynamics and transmission intensities. J Med Entomol.

[18] Molina R, Benito A, Roche J, Blanca F, Amela C, Sanchez A (1993). Baseline entomological data for a pilot malaria control program in Equatorial Guinea. J Med Entomol.

[19] Sharp BL, Ridl FC, Govender D, Kuklinski J, Kleinschmidt I (2007). Malaria vector control by indoor residual insecticide spraying on the tropical island of Bioko, Equatorial Guinea. Malar J.

[20] Hodges TK, Athrey G, Deitz KC, Overgaard HJ, Matias A, Caccone A (2013). Large fluctuations in the effective population size of the malaria mosquito *Anopheles gambiae s.s.* during vector control cycle. Evol Appl.

[21] Bradley J, Matias A, Schwabe C, Vargas D, Monti F, Nseng G (2012). Increased risks of malaria due to limited residual life of insecticide and outdoor biting versus protection by combined use of nets and indoor residual spraying on Bioko Island, Equatorial Guinea. Malar J.

[22] Kleinschmidt I, Torrez M, Schwabe C, Benavente L, Seocharan I, Jituboh D (2007). Factors influencing the effectiveness of malaria control in Bioko Island, equatorial Guinea. Am J Trop Med Hyg.

[23] Roll Back Malaria. Malaria Indicator Survey (MIS) Toolkit. Available: http://www.malariasurveys.org/toolkit.cfm Accessed October 2014.

[24] Killeen GF, Kihonda J, Lyimo E, Oketch FR, Kotas ME, Mathenge E (2006). Quantifying behavioural interactions between humans and mosquitoes: evaluating the protective efficacy of insecticidal nets against malaria transmission in rural Tanzania. BMC Infect Dis.

[25] Cook J, Kleinschmidt I, Schwabe C, Nseng G, Bousema T, Corran PH, et al. xSerological markers suggest heterogeneity of effectiveness of malaria control interventions on Bioko Island, equatorial Guinea. PLoS One. 2011;6, e25137. 10.1371/journal.pone.0025137 PMC3181341 21980386

[26] Bradley J, Monti F, Rehman AM, Schwabe C, Vargas D, Garcia G (2015). Infection importation: a key challenge to malaria elimination on Bioko Island, Equatorial Guinea. Malar J.

[27] Stata Press (2011). Stata Survey Data Reference Manual, Release 12.

[28] Rao JN, Scott AJ (1992). A simple method for the analysis of clustered binary data. Biometrics.

[29] StataCorp (2013). Stata Statistical Software: Release 13.

[30] Briet OJ, Chitnis N (2013). Effects of changing mosquito host searching behaviour on the cost effectiveness of a mass distribution of long-lasting, insecticidal nets: a modelling study. Malar J.

[31] Molineaux L, Shidrawi GR, Clarke JL, Boulzaguet JR, Ashkar TS (1979). Assessment of insecticidal impact on the malaria mosquito's vectorial capacity, from data on the man-biting rate and age-composition. Bull World Health Organ.

